# Reduced doses of dabrafenib and trametinib combination therapy for *BRAF* V600E-mutant non-small cell lung cancer prevent rhabdomyolysis and maintain tumor shrinkage: a case report

**DOI:** 10.1186/s12885-020-6626-9

**Published:** 2020-02-24

**Authors:** Yuta Adachi, Naohiro Yanagimura, Chiaki Suzuki, Sakiko Ootani, Azusa Tanimoto, Akihiro Nishiyama, Kaname Yamashita, Koushiro Ohtsubo, Shinji Takeuchi, Seiji Yano

**Affiliations:** Division of Medical Oncology, Cancer Research Institute, Kanazawa University, 13-1, Takara-machi, Kanazawa, Ishikawa 920-0934 Japan

**Keywords:** Non-small cell lung cancer, *BRAF* V600E mutation, Dabrafenib, Trametinib, Rhabdomyolysis

## Abstract

**Background:**

A *BRAF* V600E mutation is found as driver oncogene in patients with non-small cell lung cancer. Although combined treatment with dabrafenib and trametinib is highly effective, the efficacy of reduced doses of the drugs in combination therapy has not yet been reported.

**Case presentation:**

A Japanese man in his mid-sixties was diagnosed with unresectable lung adenocarcinoma and was unresponsive to cytotoxic chemotherapy and immune checkpoint inhibitors. The *BRAF* V600E mutation was detected by next generation sequencing, and the patient was subjected to treatment with dabrafenib and trametinib in combination. Although the treatment reduced the tumor size, he experienced myalgia and muscle weakness with elevated serum creatine kinase and was diagnosed with rhabdomyolysis induced by dabrafenib and trametinib. After the patient recovered from rhabdomyolysis, the treatment doses of dabrafenib and trametinib were reduced, which prevented further rhabdomyolysis and maintained tumor shrinkage.

**Conclusion:**

The reduction of the doses of dabrafenib and trametinib was effective in the treatment of *BRAF* V600E-mutant NSCLC, and also prevented the incidence of rhabdomyolysis.

## Background

Driver oncogenes such as epidermal growth factor receptor (*EGFR*)*,* anaplastic lymphoma kinase (*ALK*), and c-ros oncogene 1 (*ROS1*) have been identified in the tumors of patients with non-small cell lung cancer (NSCLC), and targeted therapy results in a longer progression-free survival than cytotoxic chemotherapy [1–3]. Mutations in the *BRAF* oncogene associated with the substitution of valine for glutamate at codon 600 (V600E) have also been detected as driver oncogene. In addition, combined therapy with the BRAF inhibitor - dabrafenib, and the MEK inhibitor - trametinib, is effective in the treatment of *BRAF* V600E-mutant NSCLC [4, 5] and was recently approved for clinical use, worldwide.

However, we observed rhabdomyolysis in response to dabrafenib and trametinib combination therapy, in a patient with *BRAF* V600E-mutant NSCLC. Therefore, we administered a reduced dose of dabrafenib and trametinib, which was able to control tumor progression, as well as inhibit the development of rhabdomyolysis.

## Case presentation

This is a case of a Japanese man in his mid-sixties referred to our hospital with lymphadenopathy of the right axillary nodes and mediastinal tumors. He was diagnosed with unresectable progressive lung adenocarcinoma (cT4N2M1c stage IVb, *EGFR* mutation (−), *ALK* fusion (−), *ROS1* rearrangement (−)). Despite multiple treatment strategies including cytotoxic chemotherapy, radiation of the mediastinal tumor, and the administration of an immune checkpoint inhibitor—pembrolizumab, the primary and metastatic lesions continued to progress. After the *BRAF* V600E mutation was detected by next generation sequencing, dabrafenib (300 mg/day) and trametinib (2 mg/day) were administered.

Initially, no adverse effects were observed, and primary lesion and lymph node metastases improved. After 2 weeks, we detected slightly elevated levels of serum creatine kinase (CK) (332 IU/L; Common Terminology Criteria for Adverse Events (CTCAE) Grade 1, normal range 62–287 IU/L), but no muscle weakness suggestive of rhabdomyolysis was observed. As a precaution, we discontinued treatment with atorvastatin in the event of rhabdomyolysis. However, after 4 weeks, during a routine follow-up, he complained about progressive myalgia and the development of muscle weakness. A serum CK level of 2247 IU/L (CTCAE Grade 3) and dark brown urine positive for occult blood were observed. A urine myoglobin level of 1400 ng/ml (normal range < 10 ng/ml) was also observed. Infection was ruled-out, and he had no history of trauma, hyperthermia, myopathies, or other diseases that could induce rhabdomyolysis; therefore, he was diagnosed with drug-induced rhabdomyolysis due to combination therapy with dabrafenib and trametinib. Intravenous fluids were administered and treatment with dabrafenib and trametinib was discontinued. This resulted in a reduction in the serum CK level, and the muscle symptoms steadily improved. This treatment strategy was effective in treating NSCLC and the patient had exhausted all other treatment options; hence, we considered continuing the combination treatment. We discussed the risks and treatment plan with the patient and his family, and they approved the resumption of therapy.

**Fig. 1:**
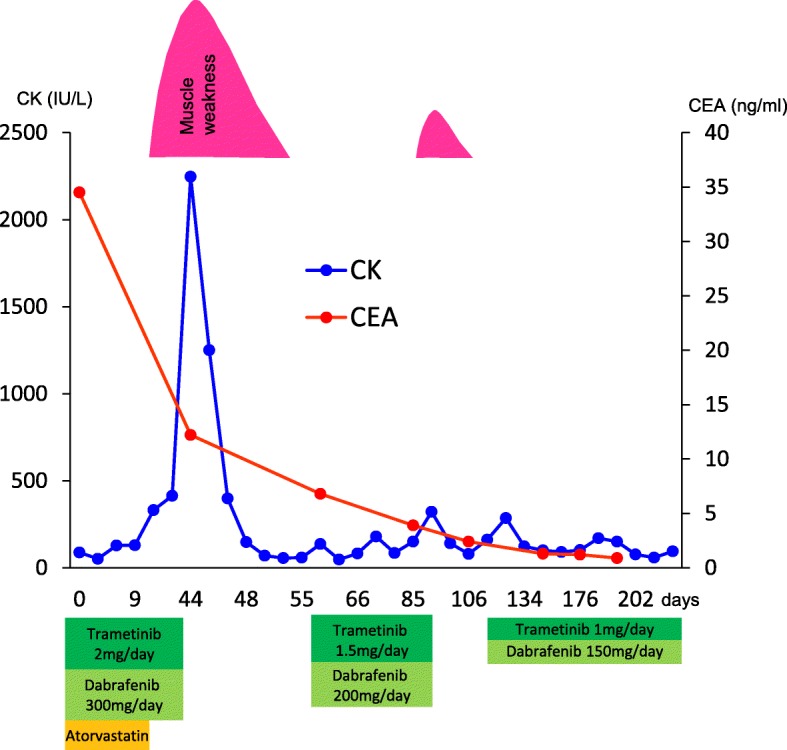
Clinical course of rhabdomyolysis and NSCLC. Reduced doses of dabrafenib and trametinib combination treatment prevented the incidence of rhabdomyolysis and tumor progression

**Fig. 2:**
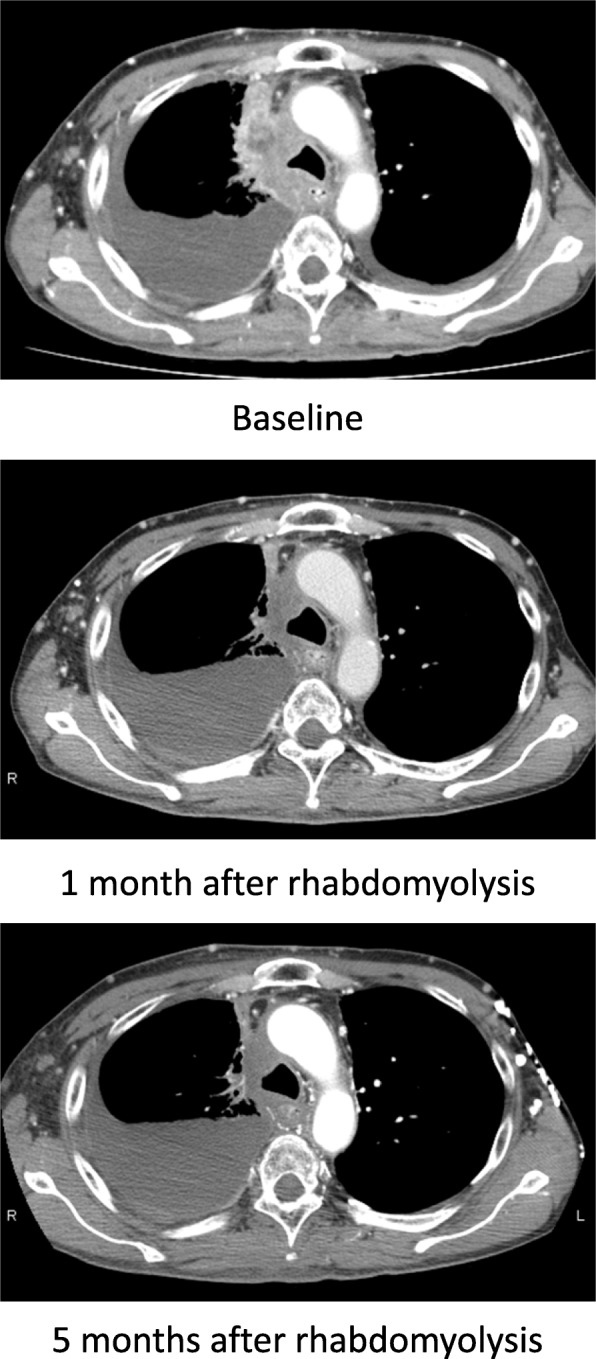
Computed tomography images of NSCLC. These images show maintained tumor shrinkage after reducing the doses of dabrafenib and trametinib

We initiated a reduced dose of dabrafenib (200 mg/day) and trametinib (1.5 mg/day) after the serum CK level returned to the normal range. No signs of rhabdomyolysis were observed until he developed muscle weakness with a slightly elevated serum CK level (321 IU/L; CTCAE Grade 1) after 1 month of being administered the combined therapy. In this instance, his symptoms and serum CK level quickly resolved with the cessation of treatment. We further reduced the doses of dabrafenib (150 mg/day) and trametinib (1 mg/day); and he has not shown any signs of rhabdomyolysis since. Primary and metastatic lesions continued to improve with the reduced doses of dabrafenib and trametinib for at least 6 months (Fig. 1, Fig. 2).

## Discussion and conclusions

We treated a patient with *BRAF* V600E-mutant NSCLC, who developed drug-induced rhabdomyolysis due to dabrafenib and trametinib combination therapy. The reduction of the doses of both drugs prevented the development of rhabdomyolysis and tumor progression. Various drugs have been reported to induce rhabdomyolysis, including statins, which are the most frequent causative agents of drug-induced rhabdomyolysis [6]. In this patient, the combination of atorvastatin or dabrafenib with trametinib resulted in rhabdomyolysis. We initially implicated atorvastatin as the causative agent because the development of rhabdomyolysis in response to dabrafenib and trametinib was not observed in the phase II trial of the treatment of NSCLC [5]. In approximately 60% of patients with statin-induced rhabdomyolysis, this adverse effect was due to the concomitant use of drugs that can inhibit Cytochrome P450 (CYP) 3A4 [7]. However, dabrafenib is primarily metabolized by CYP2C8 and CYP3A4 and trametinib is an inducer of cytochrome CYP3A [8, 9]. Therefore, dabrafenib and trametinib are not capable of inhibiting CYP3A4, which metabolizes atorvastatin.

In addition, drug metabolism is regulated by cellular transport, and various transporters are located on the cell membrane of hepatocytes, including organic anion transporting polypeptides (OATP) transporters that are necessary for multiple drug interactions including statins, which are substrates of the OATP1B1 transporter [10]. The serum concentrations of atorvastatin may increase because both dabrafenib and trametinib inhibit OATP1B1 [11]. However, in the case presented, atorvastatin was discontinued 4 weeks before the onset of rhabdomyolysis symptom. Moreover, rhabdomyolysis recurred after resuming dabrafenib and trametinib treatment. This suggests that rhabdomyolysis was induced by the dabrafenib and trametinib combination therapy, and not atorvastatin.

Rhabdomyolysis is a potentially life-threatening condition; however, the reduction of the doses of dabrafenib and trametinib may be an option for patients who experience serious adverse effects, as treatment is necessary for the control of tumor progression. In clinical trials of combined therapy with dabrafenib and trametinib for NSCLC, doses were reduced in response to various adverse events; however, the relationship between the response rate and the reduced doses has not been reported [4, 5]. One case of melanoma was reported, and rhabdomyolysis was prevented by reducing the doses of the drugs [12]. Although the relationship between the dose of causative agents and rhabdomyolysis remains elusive, one case presented the dose-dependent muscle injury induced by lenalidomide for multiple myeloma, which might suggest the cytotoxicity caused by high intracellular drug concentration [13].

We acknowledge that this is one case demonstrating the therapeutic efficacy of the reduced doses of both dabrafenib and trametinib for the treatment of NSCLC, and in order to confirm this effect and non-recurrence of rhabdomyolysis, a longer study period would be required. However, clinical trials on the efficacy of reduced doses of dabrafenib and trametinib in patients with *BRAF* V600E-mutant NSCLC are unlikely. Therefore, the reporting of real-world case experiences is necessary. Hence, reducing the doses of dabrafenib and trametinib may be a therapeutic option in patients with *BRAF* V600E-mutated NSCLC experiencing serious adverse events.

## Data Availability

The datasets used and/or analyzed during the current study are available from the corresponding author on reasonable request.

## Abbreviations

ALK: Anaplastic lymphoma kinase
CTCAE: Common Terminology Criteria for Adverse Events
CYP: Cytochrome P450
EGFR: Epidermal growth factor receptor
NSCLC: Non-small cell lung cancer
OATP: Organic anion transporting polypeptides
RO^S1^: c-ros oncogene 1
V600E: Valine for glutamate at codon 600
